# Pocket Therapeutics and Dose Book

**Published:** 1883-03

**Authors:** 


**Pocket Therapeutics and Dose Book.** By Morse Stewart, Jr., B. A., M. D. Third edition; revised and enlarged. Detroit, Mich.: Geo. D. Stewart & Co. Cloth, $1.00; Morocco, $1.50,

This little book has reached a third edition—an evidence that it is in demand. It is one of the best books of its class and contains a great deal of information, conveniently classified for ready reference and always correct and accurate so far as it goes.

